# Vaccination in Germany.—How Compulsion Is Managed

**Published:** 1899-09-30

**Authors:** 


### The Institutional Workshop.

### VACCINATION IN GERMANY.—HOW COMPULSION IS MANAGED.

In view of the discussions which have lately taken place on the subject of vaccination, and the frequent references which have been made to Germany and its vaccination laws, it may be not without interest to give an outline of the law of 1874, under which vaccination has become practically universal in Germany.

The most important clause is the first, which ordains that the following shall undergo vaccination :—

"1. Every infant before the end of the second year after birth, unless medically certified as having survived natural small-pox."

"2. Every pupil of a public educational establishment or private school, Sunday and evening schools excepted, within the year in which the twelfth year of life is completed, unless medically certified as having survived an attack of small-pox, or else as having been successfully vaccinated, during the five years previous."

There are various clauses providing for the due vaccination of those who from one cause or another may either not have been vaccinated, or may have been imperfectly vaccinated under the above clauses.

The public vaccinator shall vaccinate, free of charge, all children belonging to the district, between May and September, at times and places previously made known, and the vaccination stations must not be more than five kilometres apart.

Only medical men may vaccinate, and those who are not public vaccinators shall fill up lists and forward them to the authorities the same as the public vaccinators.

The various States will provide for the gratuitous distribution of vaccine lymph, and public vaccinators must supply lymph, so far as their supply suffices, to other medical men on request, free of charge.

A very important provision is that there shall be two forms of certificate, one stating that "by this vaccination the legal obligation has been fulfilled," and the other that "the vaccination must be repeated next year." This meets the case of those in whom vaccination "takes" imperfectly. All such cases thus get on to the list again. The certificate is in all cases the only proof of vaccination having taken place, and parents and guardians are bound under penalty to preserve these certificates, and to produce them when required.

The heads of schools have to prepare lists of pupils of an age to require revaccination. This law has now been effectually put in force for many years, and for some time back there have been very few cases of small-pox in Germany. Vaccination is not, however, compulsory in Germany, that is, if by the word "compulsory" is meant that it is done by force. The compulsion takes the form of repeated penalties if the obligations laid upon the parents and guardians is not fulfilled; but it would appear that even these penalties are inflicted for not producing the certificate when required to do so rather than directly for refusing to have their children vaccinated. It is roundabout, but effective. At the same time, the law does not override any local laws compelling vaccination in case of epidemic, and thus, in any places which have such powers under sanitary decrees, vaccination might become actually compulsory in case of an epidemic arising.

